# Religious Relationships with the Environment in a Tibetan Rural Community: Interactions and Contrasts with Popular Notions of Indigenous Environmentalism

**DOI:** 10.1007/s10745-015-9742-4

**Published:** 2015-04-12

**Authors:** Emily Woodhouse, Martin A. Mills, Philip J. K. McGowan, E. J. Milner-Gulland

**Affiliations:** Imperial College London, London, UK; University College London, London, UK; University of Aberdeen, Aberdeen, UK; Newcastle University, Newcastle, UK

**Keywords:** Buddhism, China, Conservation, Religion, Sacred sites, Tibetan Plateau

## Abstract

Representations of Green Tibetans connected to Buddhism and indigenous wisdom have been deployed by a variety of actors and persist in popular consciousness. Through interviews, participatory mapping and observation, we explored how these ideas relate to people’s notions about the natural environment in a rural community on the Eastern Tibetan plateau, in Sichuan Province, China. We found people to be orienting themselves towards the environment by means of three interlinked religious notions: (1) local gods and spirits in the landscape, which have become the focus of conservation efforts in the form of ‘sacred natural sites;’ (2) sin and karma related to killing animals and plants; (3) Buddhist moral precepts especially non-violence. We highlight the gaps between externally generated representations and local understandings, but also the dynamic, contested and plural nature of local relationships with the environment, which have been influenced and reshaped by capitalist development and commodification of natural resources, state environmental policies, and Buddhist modernist ideas.

## Introduction

Western interpretations of Buddhism often suggest that the religion is “rich in resources for ecological thinking” (Parkes 1997), reflecting an interest in Eastern philosophy in counter-culture movements of the 1960s and the idea that the world’s ecological crisis is founded in Western Judeo-Christian traditions (White 1967). This representation has perhaps been projected most forcefully onto Tibetan Buddhists, and was consolidated in the mid-1980s through the production of an environmentalist ‘Green Tibetan’ discourse by exiled Tibetan elites and their Western supporters, which was inextricably linked with nationalist politics (Huber 1997). It comes in the form of narratives about Tibetans living in harmony with their natural environment through indigenous and religious wisdom. Since that time, versions of this idea have been deployed in different ways, by a variety of interlinked actors (Yeh 2014a). Drawing upon interest in ‘sacred sites’ and indigenous knowledge as tools for conservation, interventions such as Conservation International’s Sacred Lands Program have focused on the revival of Tibetan cultural values. Chinese environmentalists and scientists have been involved in these projects since the late 1990s, whilst at the same time tourism to Tibetan areas has been heavily promoted in China, with images of a romantic and mysterious land and peoples. Tourism has been driven by state development policies to ‘Open up the West’ (*Xibu da Kaifa*) aimed at closing economic disparities between the prosperous east coast and Western provinces, and achieving political stability in Western areas dominated by ‘ethnic minorities.’ As a hotspot of development efforts, environmental conservation and religious politics, Tibetan regions, especially the borderlands of Sichuan, Yunnan, Qinghai and Gansu, have become points of intersection between different discourses and visions of nature, all with elements that are potentially conflicting or overlapping (Litzinger 2004).

We ask how these representations of ‘Green Tibetans’ relate to and play out in local understandings and practices of religion by Tibetans, using a case study of an agro-pastoralist community in Western Sichuan, on the Eastern Tibetan Plateau. Ideas about Tibetan Buddhism and the environment have tended to draw upon fantasies of Tibet and religious philosophy and doctrine rather than empirical review. Practice, however, does not always follow directly from Buddhist precept (Ramble 1990); analysis of the claim that a religion is environmentally friendly can only make sense within a particular context (Tomalin 2009). Scientific studies examining traditional Tibetan knowledge and its relationship with conservation have tended to reduce religion to binary variables, contrasting it with scientific knowledge (e.g., Shen *et al*. 2012). Instead, we use qualitative evidence from observed local discourse and practice to understand religious relationships with the environment on their own terms and in the context of social, economic and political changes. In contrast to Yeh (2014b), who focused on articulations of Tibetan environmentalism made by individuals actively involved in the environmental movement, we explore understandings across a largely rural Tibetan community that has not been the focus of major externally driven conservation efforts or community projects.

We explore the religious dimensions of people’s environmental notions specifically regarding forest use and wildlife, examining religion both as a local cosmology and as a system of moral guidance, and the relationship between these aspects. Specifically, we examine three elements of Tibetan Buddhism: (1) *local gods and spirits in the landscape* that have become the focus of conservation efforts in the form of ‘sacred natural sites’ (Wild and McLeod 2008). We examine people’s representations about local gods and their relationships with generally held norms and religious practices such as ritual and pilgrimage, and the governance of these sites; (2) *karma*: in Buddhist doctrine moral actions have karmic consequences in this life and the next. Of particular relevance to environmental issues is the precept of non-harm to living creatures. We explore how this is understood, and the relationship between karma and retribution from local gods; (3) *Buddhist moral doctrine*: Buddhism, as all religions, prescribes the best way for a person to lead their life, and Buddhist ideas regarding non-violence, moderation and interdependence have been thought to provide an ethic to deal with our current environmental crisis (Schumacher 1966; Gross 1997). Our third line of enquiry directly relates to whether Buddhist ethics in the case study community move moral consideration beyond the human world. Our analysis reflects a proposed model of ‘civil religion’ in Tibetan societies which is a composite of three parts: orthodox Buddhist ideas, cults of territorial divinities, and a secular legal constitution (Ramble 2008), the last of which is beyond the scope of this paper.

Religion is not practiced in isolation, nor does it not constitute the whole of Tibetan culture and identity. There are rapid social and economic changes occurring in the People’s Republic of China (PRC), with and within which religious ideas and authority are embedded and interacting. We situate our analysis in the context of state environmental policies implemented since 1999 and the capitalist transformations impacting the rural economy of Tibetan areas. Monasticism is a central foundation of religious life in Tibet, and is characterized by discipline, scholarship and spiritual development, as well as ritual responsibility with regard to local communities. Although our focus is on laity understandings, we examine different discourses between the monastic community and the villagers to highlight how religious and environmental understandings in the valley are related to multi-sited relationships of power with both the monastery and elites outside the local area.

## Study Site

The valley community of Samdo (Chinese: Sangdui) lies in the northern part of Daocheng County, in the Ganzi Tibetan Autonomous Prefecture, Sichuan Province, PRC, at an altitude of 3,950 m, and comprises four villages and c. 220 households. It is located in the easterly Kham region of ethnographic Tibet, the area on and around the Tibetan Plateau historically linked by ethnicity, religion and language (Richardson 1984). The majority of rural Tibetans here practice agro-pastoralism, characterised by seasonal vertical shifts to grazing land combined with permanent settlements in the valley where they grow barley. Since economic liberalisation in the early 1980s in China, demand for the parasitic medicinal caterpillar fungus (*Ophiocordyceps sinensis*) by Chinese consumers has fuelled a dramatic transition to a cash economy on the grasslands of the Plateau where the fungus grows (Winkler 2008). In 2009, on average 72 % of household income in Samdo came from selling the fungus (Woodhouse *et al*. 2014). Western Sichuan where Samdo lies forms part of the “Shangri-la Ecological Tourism Zone,” playing to the image of a Shangri-la paradise that was ‘rediscovered’ in neighbouring Yunnan in 2001 as a means of promoting tourism (Coggins and Hutchinson 2006). In Daocheng, the main attraction is Yading Nature Reserve centred on the three sacred mountains of Rigsum Gompo. In Samdo tourism is generally limited to day trips to the monastery, although there are passing tourists (mainly Han Chinese) on the road south through the valley to Yading. The community’s monastery – Bengpo – lies to the north of the valley and belongs to the Karma Kagyu school of Tibetan Buddhism. The suppression of religious practice by the Chinese state during the last century especially during the Cultural Revolution (1966–1976) and its subsequent revival means that Tibetan Buddhism is being restructured in the current political context (Goldstein 1998). Bengpo was destroyed in 1959 after Chinese forces entered Daocheng in 1951, and rebuilt after liberalization in 1982.

Western Sichuan Province is recognized by conservation organizations for its high level of biodiversity and endemism (Conservation International 2012). In the wake of devastating flooding in the Yangtze River Basin in 1998, a shift in forestry policy was made from timber production to planting and conserving forests with a series of environmental programmes under the auspices of the ‘Open up the West’ policy, which combines major investments in infrastructure development, education, and mineral exploitation with environmental protection and restoration (McNally 2004). Hunting has been prohibited in Daocheng since 1989 under the Wildlife Protection Law. Conservation work has been carried out by the World Pheasant Association (WPA), which was drawn to the locality by the threatened population of white eared-pheasants (*Crossoptilon crossoptilon*) fed daily by monks in Bengpo monastery. A Chinese ecologist associated with the organisation, alongside several students, have carried out ecological surveys and implemented small-scale education activities. The research upon which this paper is based was itself instigated through WPA due to interest in the potential for Tibetan culture to align with conservation goals.

## Methods

**Fig. 1:**
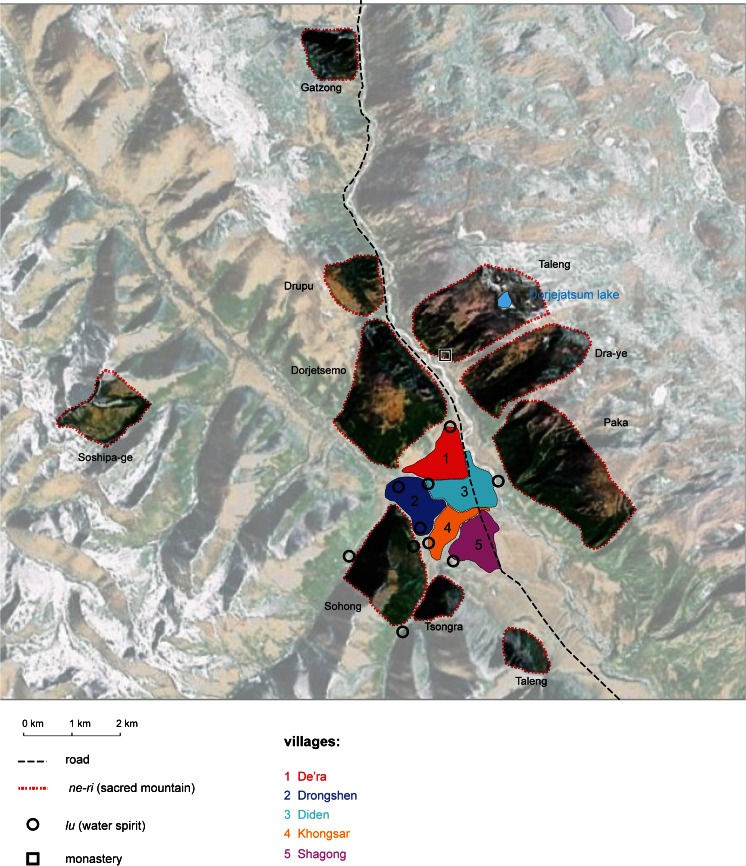
Map of Samdo showing local gods and water spirits. Satellite image from Google Earth © CNES/SPOT 2012

We used a combination of mapping, household and key informant interviews, and participant observation to collect qualitative data on religious ideas about the environment, environmental policies, the cultural landscape, ritual practices towards local gods, and livelihoods. In June 2009 we carried out a participatory mapping exercise involving five men (including one monk), facilitated by a Tibetan interpreter, with the aim of exploring (1) locations of and characteristics of sacred land; (2) local perceptions of and practices towards the environment; (3) customary land use, boundaries, and areas of resource extraction. Due to cultural difficulties of including women, only men participated in this activity. The resulting map was, however, discussed with women during semi-structured, key informant, and informal interviews to ensure their perspectives were understood and represented. Community members volunteered to locate areas identified in the map, and the co-ordinates were recorded with a global positioning system (GPS), thus integrating local knowledge into a geo-referenced map using satellite imagery (Fig. 1). The maps were used as a basis for further discussion during household and key informant interviews.

Semi-structured household interviews were carried out during January–April 2010. The household is the unit of economic activity including natural resource use as well as ritual practice, so interviews were conducted at this level with the head of household or those involved in household decision-making (including women). We selected households by numbering all the houses in the valley, selecting a random number and visiting the corresponding house. Open-ended questions and discussion were used to provide flexibility. The questions were piloted in six households in a neighbouring valley and then 50 interviews were conducted in Samdo, one in each household sampled. Interviews were carried out in Kham Tibetan in the respondents’ homes with help from a translator, recorded in agreement with the participants, and translated into English.

During the period of field work (May–June 2009; January to April; August to December 2010) we gained further in-depth knowledge through discussions with people in the community (including monks, local government workers, and elders) who became key informants. EW lived in the community during this time, providing opportunities to observe everyday situations and allowing contextualization of the interview data. Interview data were stored, managed and coded using NVivo (QSR International 2010). Analysis involved sorting data to identify common sequences, phrases, patterns and themes, ensuring comprehensive treatment of the data.

Fieldwork began just after a period of heightened security and restrictions on freedom in response to Tibetan protests during spring of 2008. Official authorisation and relations with local government through alignment with WPA allowed relative freedom to carry out the research. However, some people (both laity and monastic communities) were at times fearful of reprisals for speaking to a foreign researcher, especially regarding formal religious activities, which lie under the state’s control, and mountain deities, which can be labelled as superstitious (*mixin*). Building up relationships and trust through time with key informants allowed for access to knowledge and practice, but there were unavoidable limitations to the depth of data obtained through household interviews.

## Local Gods in the Landscape

On arriving in a Tibetan village it is obvious that religion plays a part in everyday life: local people spin prayer wheels whilst they chat, prayer flags flap in the wind and *chorten* - architectural structures holding religious relics - dot the landscape. What is not immediately apparent is the world of gods and spirits that also forms part of this landscape for Tibetans.

Tibetan Buddhism has inherited several models of the universe from Indian Buddhism, one of which depicts all forms of existence in the ‘Wheel of Life.’ The motif, commonly seen in monasteries, shows all beings circling in the six realms of *samsara* (the unsatisfactory cycle of death and rebirth) including gods (*lha*), humans, animals, hungry ghosts, and the hot and cold hells. Within this formalized structure there exists a local cosmology in Samdo as in all Tibetan communities, containing a variety of gods, spirits and ghosts. Local or regional gods are distinguished from supra-worldly gods (*jigten le de pe-lha*), who have moved up to the heavens through their own efforts in acquiring merit, and the Tantric gods of Buddhist meditation (Samuel 1993). Local lha remain ‘gods of this world’ (*jigten pe-lha*), although the distinction can be inexact. Although representations of these gods are localized there is a widely recognized typology described by scholars of Tibet (Mumford 1989; Samuel 1993; Mills 2003). In Samdo, the main types of local gods and spirits are:

*Yul-lha* - territorial deities most associated with mountain domains overlooking the villages (*yul* means local area). They are dominant in the religious landscape of Samdo and thus the main focus of discussion here. Local people commonly identified and named ten different yul-lha surrounding the valley (Fig. 1).

*Lu* – water spirits whose physical appearance is half human and half snake; they belong to the underworld beneath the domain of humans and live in water, sleeping in their shrines in the winter and awaking in spring time. They are mainly associated with fertility and considered capricious.

*Sa-dak* – earth spirits or owners (*dak* translates directly as ‘owner’) associated with more localised natural features such as rocks, stones and small areas of land. Local people also referred to *chu-dak* (water owner), *shing-dak* (wood owner) and more generally *zhi-dak* (foundation owner) based in mountain abodes, and a term sometimes used instead of yul-lha.

### Ritual Observance

Ritual actions performed towards the gods form an important way of defining relationships between humans and other beings in the landscape. In Samdo, the shrine (*lha-to*) to the local god is often near the base of the mountain on another small hill. Villagers (men and women) go to their nearest or the village yul-lha shrine to make offerings, burn incense, and hang prayer flags (*longta* meaning ‘wind horses’). This is generally a biannual tradition performed at Tibetan New Year (*Losar*) and before the harvest in the ninth month of the Tibetan calendar, but also on any auspicious dates or whenever people need the deities’ help. This ritual observance constitutes part of the calendar of regular rites within the household and monastery. Ritual practice takes place on a continuum from the personal and domestic, for example on daily basis in the home with regard to household gods and protective deities (often carried out by women), to exterior spaces of the fields, forest and mountains.

Ritual attention to the water spirits, lu, regarded as quite different beings, consists of offerings of milk, wool, tree branches and barley made in June and July of the lunar calendar, after the lu awake. One, named Bemalen, is the most significant for the whole valley; others are more locally important and historically associated with particular lineages and groups of houses. Senior clerics at the monastery are also involved in ritual practice towards the local deities. Using a supra-worldly god invoked through the use of tantric practice, they order the yul*-*lha to protect people and not to punish them. This is a re-enactment of the subduing of the worldly gods, a common theme in Tibetan Buddhism, recalling the initial control exerted over the landscape by the incoming religion (Ramble 1999). In addition, every day, a monk chants scriptures in the ‘protector temple’ (*gon-khang*) of Bengpo monastery and devotes a few minutes to entreating the worldly gods to protect the earth from natural disasters.

By far the most important yul-lha in Samdo, the territorial deity for the whole valley, is named Dorjetsemo. Every Tibetan household interviewed spoke of it as their yul-lha, and many people (28 of the 50 households surveyed) said they had circum-ambulated its mountain domain (approximately 10 km) and made offerings to it in the previous year (2009). Dorjetsemo is considered the leader of all the lesser deities in the valley. Dorjetsemo, like other indigenous gods, may have found its way into the formal Buddhist pantheon through the use of the word *dorje* meaning ‘diamond,’ and given auxiliary functions by acting as emanations of a god or guardians of the ‘doors’ to these gods (Tucci 1988). Indeed, Dorjetsemo is allied with another much more famous pilgrimage site further south in Daocheng county – the peaks of Rigsum Gompo at Yading - the three protector bodhisattas (beings motivated by compassion to be dedicated to attaining enlightenment).

Dorjetsemo forms part of a popular pilgrimage for people of Samdo and villages beyond, encompassing two other mountains and a sacred lake. These sites were thought to hold particular power due to their history; for instance Dorjetsemo has a precedent of important lamas meditating there. People go on pilgrimage to these sites to gain merit and blessing (*chinlab*) through ritual acts. Buffetrille (1998) describes two types of sacred mountain in Tibet: that of the locally relevant yul-lha; and those worthy of circumambulation (*ne-ri*), which have been converted into Buddhist supra-worldly divinities through a process of taming (*dul-ba*) significant to the wider Tibetan community. *Ne* are the foci of pilgrimage and can be a variety of objects including religious structures and incarnate lamas. With the word mountain (*ri*) ne denotes the mountain abode of a regional god (Huber 1999a). Local Tibetans in Samdo in fact often use the words ne-ri and yul-lha interchangeably (although the monastic community make the distinction), and some respondents recalled circum-ambulating another yul-lha – Sohong. This suggests that the significance of a local god can be dynamic and pilgrimage to a particular place may wax and wane accordingly (see also Wenbin 1998).

Households perform rituals for certain gods, especially those closest to their village, more often, reflecting the symbolic identification of villages with local deities in Tibetan communities (Pirie 2006). Informants discussed the relationship between particular villages and yul-lha: the second village (Drongshen) is associated with Sohong, and the fifth (Shagong) with Tsongra and Taleng. Dorjetsemo and Gatzong have ritual significance to all villages, and beyond to the neighbouring valley from which people come to circum-ambulate the mountains. The yul-lha Soshipa-ge, which lies far from the centre of the valley, was mentioned by only 15 of 50 households surveyed (Fig. 1).

### Environmental Norms

Conservationists have suggested that sacred sites are akin to protected areas and represent a form of informal institution for natural resource governance that may incorporate non-extractive norms and active protection and management by local custodians (Wild and McLeod 2008). Indeed, sacred forest in Tibet has enhanced tree size and cover in comparison to surrounding areas (Salick *et al*. 2007). The concept of the sacred in conservation tends to follow Emile Durkheim’s (2002) definition, as that which is “set aside and forbidden” and in direct opposition to the profane and every-day. In Samdo, non-extractive norms are related to indigenous knowledge of the local god’s location, and fears of retribution, which potentially lead to protection of specific sites. The community unanimously agreed that people should not cut trees or plants, dig earth and stones, or kill animals on the yul-lha, some adding other misdemeanours such as making fires, shouting, urinating and fighting. Similarly there are strict norms about cutting trees and digging earth near lu. For sa-dak and other more geographically confined spirits, norms are less often evoked due to the uncertainty concerning their location and their ubiquity, and are more likely to be overridden by practical concerns.

Breaking the norms associated with gods and spirits in the landscape is directly associated with misfortune, most commonly sickness. Symptoms, especially visible signs that carry social stigma, rather than specific diseases are important, e.g., skin lesions and boils, pain in the joints and limbs, and the loss of hair and eyebrows, all of which can be related to leprosy (*dze*), which was sometimes referred to explicitly and is often connected with ritual pollution and retribution from deities (Mumford 1989). Numerous other misfortunes related to breaking norms were also discussed: bad weather (floods, storms and rock fall), poor agricultural production (bad harvests, wolves attacking livestock), and general bad luck. Conversely, positive norms to protect the god bring luck, health, and a good harvest. Misfortune is directed at the individual norm breaker (although this is sometimes extended to family members and hereditary diseases). However, in the case of a more general scenario of a local god’s forest being cleared on and the culprit not specified, respondents discussed how retribution could fall on all the villagers, shortening life expectancy and decreasing fertility of crops and livestock. The gods are described as embodied within the landscape, reflecting the tendency to anthropomorphize the landscape. There is a perceived connection between the local gods and the wellbeing of the valley as a whole.

“The local gods are like people – they have flesh, bones and a heart - so when you cut down trees it is like taking part of their body.”

*The meditation leader (Drupen), Bengpo monastery, 2009*

Huber (1999b), for example, notes how killing animals at the mouth of a goddess embodied in a mountain was said to please her. The contours of the land are conceived as body parts of animals or human-like beings. Dorjetesmo is in the form of a white deity riding a white horse which can become an untamed tiger when angry. Both the deities themselves and the rituals associated with them were also gendered. Sohong, for example, is female and associated with enhancing (a particularly feminine form of) beauty, and only women perform her rituals. Only men climb to the top of Dorjestemo at Losar to place prayer flags at dawn, although both men and women circumambulate it. Inappropriate conduct through subverting these gender norms is said to lead to hail and general misfortune.

Interviews revealed inconsistent and contested norms in relation to representations about the inhabitants of the landscape, their history and personalities, and more worldly concerns. Respondents consistently described the different temperaments of each god, which can be broadly separated into wild and unpredictable or benign and helpful. Gatzong is particularly ferocious and threatening, and his punishment will happen quickly and forcefully, whereas Dorjetsemo as the leader of the lesser deities is more benevolent and is compared to a monk (*gelong*) who had taken vows of Buddhism (*dompa*). All the yul-lha, however, can punish if norms are broken. Particular incidents were consistently recalled: a man tried to cut down trees on Gatzong and instead cut off his own feet; in 1990 removal of stones for building from Sohong despite warnings by the monks and local community, was said to have resulted in the car transporting the rocks to crash. The religious significance attributed to (and concern about angering) Sohong seemed to have shifted as a result.

Respondents reported how they were unable to protect the gods during China’s Cultural Revolution (1966–1976). Explanations included material rationales: extreme poverty, and in particular the need to earn money from selling charcoal from local trees, as well as religious reasons: that the gods had escaped with Tibetan refugees across the Himalayas to India from the late 1950s when “democratic reforms” in the PRC were implemented, only returning when religion was once again politically acceptable from the early 1980s. Norms changed as a result, so that cutting down trees on the yul-lha became admissible. The conditions of social chaos and violence during this time highlight ‘quandaries of agency’ for Tibetans (Makley 2007). The return of deities and renewed adherence to religious norms were a means of signalling Buddhist devotion as well as Tibetan identity, marking a distinction from the Maoist period of moral uncertainty. Highlighting this, on being asked what would happen if trees were cut down on a yul-lha, one lay man simply replied “That would be destroying Tibetan *la-ja*” – a word that denotes honour in and loyalty to a culture.

The negotiation of moral dilemmas is also evident in the emergent demand for caterpillar fungus. Older people in Samdo remember a time when it was taboo to collect the fungus on yul-lha or in fact any land, because digging the earth kills insects – a sin - and may disturb earth spirits. Despite this, many people insisted that there were no restrictions on collecting the fungus, or justified the extensive collection as “the only way to get a high income here,” reflecting how the fungus has improved rural lives, and the competitive disadvantage many rural Tibetans face in employment due to disparities in education and skills (Fischer 2005).

The boundaries of sacred land are uncertain and flexible. The yul-lha do not necessarily equate to a whole mountain, and the face of the mountain not visible from the village is not considered part of the god. Only the forested section of the mountain is regarded as the yul-lha Tsongra, suggesting that forest is significant in the recognition or development of the sacred site, perhaps because trees and plants are considered adornments of the gods. In Samdo, the higher reaches were indicative of the gods’ power, reflecting the tripartite distinction in the cosmological ordering of the Tibetan landscape in which the lha occupy the highest level and the water spirits the lowest (Mills 2003). Respondents indicated that firewood cutting and removal of earth to make walls were acceptable towards the base of the mountain (see also Weckerle *et al*. 2006). Most people are unsure of the boundaries of specific yul-lha but generally consider them to be natural features such as valleys and rivers, suggesting ‘gradients of sanctity’ rather than defined limits (Salick *et al*. 2007). Local people are also uncertain about the locations of the sa-dak so that care is required when digging the earth, for example performing rituals before building houses.

The yul-lha are, on the other hand, well-known and associated with the valley where they reside, as a senior cleric explained, “like each shop has a keeper, each yul-lha belongs to a place….he is responsible for the lives near to him.” However, the presence of yul-lha at some sites, most significantly the ‘*Paka*’ (the ‘other side’ mountain) on the east side of the river is contested within the community, with some people (16/50) recognizing it as a god and others not. Respondents indicated some flexibility in norms under certain situations, for example the need to make fires and collecting caterpillar fungus. This supports a situational definition of sacredness in which nothing is inherently sacred, but rather sacredness is produced and defined through the ritual activities. In Samdo, the flexibility of norms also reflects the ‘lay dilemma’ in Tibetan communities (Mumford 1989) that entails people accepting a certain amount of sin or punishment for their everyday actions.

## Protecting our Valley: the Territoriality of Local Deities and Land Governance

Many people in Samdo consider themselves the owners of the material mountain body of the local gods rather than the state (which officially owns the majority of land in the township). In the reciprocal relationship the villagers have with their local god, they ‘protect’ it by praying and making offerings rather than in any physical sense. Some people also emphasised that the yul-lha itself was the owner, using the word *dakpo*, which denotes having power or jurisdiction over particular areas (Mills 2003). Respondents reported that they only physically protect the land from outsiders (including Tibetans and Han Chinese from outside the valley), rather than members of their own community, highlighting how the deities are intimately linked with, and define the boundaries of the community as a political entity:

There are no owners [of the yul-lha] - all the people in the valley are the owners and protectors. If someone from another area like Dapba [Daocheng] town or Chatreng [Xiangcheng] comes, then all the people will stop them from killing animals and cutting trees in this area – on the yul-lha and the other mountains. We don’t stop people from here, only people from other places. All Tibetan people only pray for the yul-lha, they don’t stop people from doing things on it. People don’t cut trees and kill in other areas because they are afraid of a fight – it is dangerous. It is our responsibility to protect our own area…the government has not dealt with the boundary problems. [*38 year old layman, pastoralist*]

The religious landscape defines the contours of the valley and its sub-communities. Dorjestemo is particularly important in this respect; as the principal mountain deity it holds sovereignty over the communal territory. Expressions of territory over land were made most vociferously with reference to access to caterpillar fungus on the grasslands which triggered violent conflict between Samdo and a neighbouring community in 2007. In Rebgong, Makley (2013) found that the revival of rituals associated with a mountain deity was at the centre of contests over land ownership and political representation in the face of state mandated urbanisation. In Samdo local gods are said to fight with yul-lha in the surrounding area, echoing human conflict between different valleys. Here, recognition of local gods as alternative authorities to the state asserts Tibetan local autonomy and cultural identity, and also defines communal territory where Tibetans reclaimed authority in the face of perceived government failure to define boundaries and protect a livelihood source now intimately tied to rural Tibetan lives.

No examples of actions taken against members of their own community who had broken norms on the mountains of local gods were given. Speaking about a hypothetical situation in which someone was cutting trees on a yul-lha, one woman said:

I would try to persuade the person. If they didn’t listen, I wouldn’t do anything. It would result in them getting sick. On other land, I wouldn’t say anything because the Forestry Bureau is in charge there. [*50 year old woman; agro-pastoralist*]

The local gods are regarded as arbiters of moral justice on specific lands that represent ancestral heritage for local people. On the other areas, excluding boundary areas where the stakes are high due to the presence of caterpillar fungus, state authority is generally accepted as legitimate.

## Karma and Environmental Actions

### Non-violence and Good Fortune

The explicit goal of Buddhism is enlightenment, transcending the suffering of the normal cycle of rebirth (*samsara*), to gain understanding of the true nature of reality. The movement between births as different beings in the ‘Wheel of Existence’ is not random but governed by the natural law of karma (*lé*) related to actions, the effects of which can also be felt in this life. Respondents commonly referred to the concepts of fortune (*sonam*) and sin (*dikpa*) with regards to karma and morality, and mainly with reference to this life rather than a fortuitous rebirth. *Sonam* can be described as ‘ethical power’ which increases with virtuous deeds, can be exhausted, and is part of a ‘body of fortune’ which is essentially unknowable so that immediate good fortune e.g. increases in wealth may end in negative consequences (Da Col 2007). This perspective is reflected in Samdo in expressions of unpredictability in the world, and sometimes pragmatic resignation about the inevitability of committing sinful acts. With regard to the environment, karma is associated with the first precept in the ten Buddhist prohibitions – not taking life, especially the hunting of wild animals. In Tibet it is a common practice (named *tse-thar* - ‘free a life’) to release animals, especially fish, chickens and cattle, from captivity to escape slaughter in the hope that the animal will return the favour in a future life. The reciprocal relationship between beings in the cycle of rebirth spans multiple future lives.

Unlike actions related to harming local gods where even an accidental transgression can lead to punishment, motive is considered important with regards to karmic sin and good fortune. An unintentional sinful act will not exhaust sonam to such a degree and is more easily remedied through ritual action. Accidental or necessary taking of life is justified by practical realities:

In the fields there are so many insects, so people kill them too. When they eat tsampa [barley meal] from their hand, blood will drop from their fist. If you dig the ground you will get a lot of sin, but if you don’t dig it, you won’t have any food to eat or clothes to wear. [*52 year old man; agro-pastoralist*]

This imagery clearly shows that insects fall under the moral sphere of karma, and to a certain degree this concern extends to plants too. Despite the ambiguity around the moral significance of plants in Buddhist doctrine, many respondents consider them and especially trees to “have a life” (*tse-sok*) and therefore it is sinful to kill them. There are some references in Buddhist texts to bad karmic consequences of cutting trees, and the benefits of planting groves (Keown 1992). The moral worth placed on plants in Tibet is addressed in environmental discourse amongst Tibetan religious elites and particularly incarnate lamas, regarding the morality of cutting down trees (e.g., Tenzin Gyatso 1993; Ogyen Trinley Dorje 2011). Reference to teachings regarding hunting and deforestation connected to the Dalai Lama and the Karmapa (who is revered in Samdo as the head of their school of Tibetan Buddhism) in particular are common amongst the laity. This appears to be a relatively recent development in Samdo, as older people recalled that there were no religious restrictions on cutting trees on land not governed by the monastery or the domain of a deity.

Respondents indicated that improvements in forest during the last decade result from both religious resurgence since liberalisation and government forest protection policies, often presented as mutually reinforcing. This reflects progression in Green Tibetan discourse from opposition to the state to a strategic alignment with state policies (Yeh 2014a), which since 1999 have become more environmentally protective. Although state policies were largely presented through a religious lens, religious and scientific rationales (e.g., soil erosion) for protecting forest were also not always presented separately in discussions suggesting the pervasive nature of state discourse.

### Interactions Between Karmic and Local Deity Models of Fortune

As karma is a natural law, doctrinally it is not considered to be related to the retribution dispensed by the gods but rather all beings in existence are subject to it, including worldly deities:

If you cut trees, you will get leprosy afterwards, or immediately ache and die at the same moment. If you kill animals on the yul-lha, you will get the same result. If you did these things where there is no yul-lha, you will get sins but no punishment from the gods. [*60 year old man; agro-pastoralist*]

For some people, however, there is little difference between the results of killing animals or cutting trees anywhere; only the degree of misfortune suffered rather than the type is distinguished. The function of local gods in Tibet can include “increasing the possibilities of good karma” although enlightenment is beyond the reaches of their powers (Tucci 1988). In Samdo, the harming of gods was not discussed in obviously ethical terms; the word sin was not used in reference to it and only consequences were focused upon. Yet actions related to supernatural beings, in addition to virtue, can stave off bad karmic consequences. In terms of ideas in conservation about sacred sites, it also further narrows the distinction between the sacred and other areas of land.

## Buddhist Precepts: an Environmental Ethic?

Western interpretations of Buddhism and Green Tibetan representations propose a more intimate relationship between religious precept and environmental concern and action than one based on egotistical fear of consequence. In Tibet, the concept of *tsul-trim* refers to moral discipline, and is an intrinsic element of the path to enlightenment. In Tibetan Buddhism moral guidance comes mainly in the form of the ‘ten non-virtues’ or prohibitions, the first of which, as already discussed, is taking life. During discussions about sin, respondents did not always seem to be connecting moral action with karmic consequences in this life and the next. Killing was not necessarily related to material consequence but simply “bad in Dharma” (*chö*). In fact the importance of karmic outcomes and Buddhist doctrine are not mutually exclusive and form part of the same moral order; Dharma - the Buddha’s teachings - is manifest in the law of karma which governs the way moral deeds affect individuals. In this way sonam (luck) can be understood as an experiential indicator of moral virtue, instead of a rather crass system of rewards and punishments for good and bad behaviour (Keown 1992). In addition, the word ‘happiness’ (*tsi-po*) in connection to karmic good luck, often used by respondents, is not purely egotistical but is mainly evoked in a communal sense. Through karma, the idea of happiness incorporates material good fortune *and* virtue; a happy life is not only obtained through virtue, but constituted by virtuous actions. There is no real dispute between following a strong moral precept and holding the karmic effects of some consequence.

Happiness is considered directly related to the state of the environment – the number of animals and size and health of the forest – which is generally thought to have improved in recent years. There was some ambiguity regarding the direction of cause and effect of this improvement – with religious resurgence and therefore virtuous actions leading to a better environment, and virtuous actions also including protecting animals and trees, so that relationships between sonam and the environment were reinforcing. Critiques of Western environmentalism by Tibetan activists hold the ultimate cause of environmental destruction to be sonam, dependent on people’s hearts, rather than proximate causes of logging and pollution (Yeh 2014a). There is also a historical and political foundation to this idea intertwined with religious identity. The hunting of animals appears frequently in historical narratives about the Cultural Revolution, when religious activity was prohibited, and resurgence in religious practice is directly associated with the ability to protect forest and wildlife (although again here also facilitated by state policy):

When I was in my 20s I killed many animals, I had a gun then. The government had said that there are no gods or ghosts and no such thing as sin, so I killed everything except people! If people have religion (*chö*), then they don’t kill. When I was in my 30s I regretted what I had done, and didn’t kill any more…Before the environmental protection laws, people’s lives were very poor…they were dying of starvation so they had to kill animals. [*57 year old man; agro-pastoralist*]

“No-one hunts now” was a stock response from respondents. Some people, all older men who had hunted and saw its benefits, however, provided more pragmatic perspectives, stating that they would hunt animals if the government had not confiscated their guns. This perhaps reflects a long history of hunting on the Tibetan Plateau (Huber 2005), resentment of government policy restricting autonomy, as well as an emphasis in Buddhist modernist discourse on hunting and environmental protection that has been taken up by younger Tibetans who have never hunted.

The Buddhist concept of ‘dependent arising’ that postulates that all phenomena arise, remain and cease in relation to everything has been translated in Western Buddhism as an ecological idea of interconnection between all organisms, an idea also drawn upon in the environmentalism of Tibetans religious elites (e.g., Ogyen Trinley Dorje 2011). Although this interpretation is not accepted in the Samdo community it does hold some relevance. Certainly, respondents openly stated their empathy towards wildlife, indicating that animals merit moral consideration and are part of the same system as humans (“they suffer just like humans”) and in particular that they are reincarnate – indeed, they once may have been human as humans may once have been animals. As Pirie (2006) noted, Buddhist principles are generally not invoked with regards to human relationships but rather decisions are based on desirable community benefits. In relations with non-human animals, however, religious sin was significant in discussions in Samdo. Respondents nonetheless made a definite demarcation between humans and animals, the former being more fortunate with increased sensory skills. Similar feelings of compassion or anthropomorphism were not expressed about plants, reflective of traditional doctrine that plants are not in rebirth cycle – you cannot be reborn as a plant.

Based on Buddhist precepts, pre-1950 Tibetans killed fewer larger animals and there were taboos about hunting certain animals such as foxes based on morphology (Huber 2005). In Samdo less value is placed on small animals, insects and domestic animals, but beyond this all wild fauna is considered broadly equal. It is important to note that concern was expressed over the suffering and death of individual animals rather than about species or populations, although there was recognition of the decline in numbers of some species. This raises doubts about whether Buddhist ethics could support modern conservation principles, since the protection of threatened species as abstract entities does not align with Buddhist respect for individuals (James 2006).

The idea of renunciation or moderation in wealth also has potential implications for natural resource accumulation and use. Schumacher (1966) argues that Buddhists do not become attached to wealth, which stands in the way of liberation, but rather satisfy their needs by means of modest use of resources. From a conservation perspective, modernity and the global capitalist economy are often seen as a threat to indigenous beliefs connected to environmental sustainability (Ormsby and Bhagwat 2010). In Samdo access to cash has increased dramatically over the last 10 years, fuelled mainly by the trade in caterpillar fungus. People are investing their new-found wealth in housing, motorbikes, and electrical goods. House size is fiercely competitive and exemplifies ideas of tradition and cultural identity - houses are often large and ornately decorated inside with traditional cabinets. There was no discussion of the environmental implications of resource use associated with building a house by either lay or monastic respondents, which may reflect the fact that timber is collected in a different valley so environmental impacts are remote and not routinely visible. Monastic discourse regarding moderation reflects ‘Green Tibetan’ ideas regarding the morality of wealth accumulation (Ogyen Trinley Dorje 2011), and there is some evidence of reflection on these teachings and ideas about their own consumptive behaviour by the laity:

The lamas always say that working hard to build houses is not good. If you have a house that is good enough, and it is better to have clothes and food, but people don’t listen… [*68 year old layman; agro-pastoralist*].

Increase in consumption does not necessarily suggest that Tibetans have become more concerned with displays of wealth, merely that they now have greater access to consumer goods. Although there may be a moral concern for individual asceticism in Tibet, especially for monks, this does not necessarily preclude a concern for wealth and prosperity at the household level and within ceremonial life (Mills 2006). In fact, the protection of health and prosperity is a focus of many rites and perceived as the product of karmic good fortune. The way in which wealth is attained and used is a concern, however, illustrated by the consistent view of the laity that housing is a socially correct use of income, whereas habits such as drinking alcohol, smoking and gambling are improper and a waste of money. These behaviours, along with meat eating and the trade in animal pelts, have been targeted by modernist movements across the Tibetan Plateau, which encourage the laity to take oaths not to engage in them. On the other hand wealth creation supports religious resurgence, for example the construction of religious monuments, monastery sponsorship, and pilgrimage to important Buddhist sites. There is a connection between religious virtue and wealth in that many obvious forms of action connected to merit-making require significant financial resources (Samuel 1993).

## Differences Between Lay and Monastic Discourses

Spiro (1970) suggests two distinct models in Buddhism, pursued by the laity and the monastery, which echoes treatments of Tibetan Buddhism which present a lesser ‘folk religion’ containing local gods and spirits as violating a pure philosophical tradition. This effectively ignores the ritual role played by clerics, which is the basis of religious authority and is embedded within local cosmologies (Mills 2003). The religious worlds of the laity and monastery are integrated in Samdo but each group spoke about religion in different ways. With regards to local gods, senior monks made a clear distinction between the ‘real gods’ of Buddhism and the local worldly gods whose capabilities they dismissed, despite their ritual relationships. The Dalai Lama himself has publicly renounced spirit worship as superstition, rather promoting more morally based Buddhist ideas in line with Buddhist modernism (Pirie 2006). This distinction was not something emphasized by our lay respondents, who spoke about religion from a more practical perspective, although within a moral framework. Enlightenment – the ultimate goal of the Buddhist path – was not referred to explicitly by the laity, only by monks, which suggests differences in the way these groups frame the moral elements of Buddhism. For example, the monks connected norm breaking on the yul-lha with morality (non-violence and compassion for others) and the authority of lamas rather than potential consequences.

The monastic community more directly and purposefully draw upon elements of ‘Green Tibetan’ discourse in their discussions of religion and the environment, especially regarding idealised historical narratives regarding pre-Maoist Tibet in which religion flourished and thus the environment was protected. Several of the higher level clergy have travelled to India where they would have had direct exposure to these ideas, and have access to the internet. Although the lay respondents reflected on teachings related to this discourse, and they asserted their Tibetan identity and community autonomy by religious practice especially regarding local gods, any links to the environment were less self-consciously made. Indeed, they generally had no understanding of and/or interest in the ecological work of WPA, which largely works in collaboration with the monastery, perhaps because it was perceived as externally led, did not hold religious authority, or chime with local priorities.

## Conclusion

This research highlights the contrast between religiously oriented understandings of the environment and Green Buddhist representations in their various guises, where they intersect, and how elements of Green Tibetan discourse are being articulated and reshaped in one rural locality. In global conservation, sacred sites are perceived as conceptually and materially distinct from other areas. Although there were strong non-extractive norms and ritual attention paid towards the local gods, they exist within the lived experience of local Tibetans, so that nature is not something external but a part of the social world. Boundaries of local gods were uncertain and there is differential ritual adherence to particular gods, so that the sacred is constituted by relationships between specific groups of people and specific supernatural beings. These relationships are dynamic, dependent on social context and linked to social memory of unfortunate events. Protection of local gods is stated in ritual terms, and resurgence in devotion to these mountain gods asserts cultural identity and autonomy, defining local territory especially with regard to inter-community relations soured by disputes over access to caterpillar fungus. The concepts of sin and karmic retribution are also intertwined in this model in understandings of misfortune, further obscuring the concept of the sacred as a category related to distinct sets of non-extractive behaviours. The focus on proximate consequences of actions and worldly goals by the laity did not preclude a moral understanding of relations with the natural world. Interrelatedness was expressed as an anthropomorphic expression of affinity with living organisms within a graduated scheme of value rather than either an eco-centric or human-centric ethic.

We highlighted the dynamic nature of perceptions and practices towards the environment. As the highly valuable caterpillar fungus has become economically significant it has been negotiated into a morally acceptable form of natural resource extraction. Rather than comprehensively embracing capitalist development and materialism since economic liberalisation, however, norms regarding wealth reflect historical attitudes, ideas of social identity, and echo Tibetan Buddhist modernist discourse resistant to secular state ideology. This discourse has also influenced lay conceptions about the sinfulness of killing trees and plants. There is not a mutually exclusive relationship between following religious and government laws, with Tibetans engaging with and interpreting state discourse and policy through a religious lens. Overall, there is a plurality of values towards nature reflected in particular distinctions in the discursive domains between the laity and monastery. In both, local cosmology, Buddhist precepts, and contemporary materialistic views, are pieced together in different configurations and related to salient aspects of people’s lives – a pattern that Salick *et al*. (2012) also found in Tibetan interpretations of climate change.

Interest in religion and indigenous culture fulfils contemporary conservations goals of grassroots participation and socio-cultural legitimacy, but the complexities described here indicate that a nexus between local culture and modern conservation may be elusive. However, modern environmentalist discourse – especially as represented in Buddhist modernist teachings and through the authority of religious elites – can potentially reinvigorate local religious ideas and practice with positive environmental impacts. Knowledge about the environment is changing in Samdo, is reinterpreted through government ideology and Green Tibetan discourse, and influenced by cultural politics. The last decade has witnessed Tibetan-led environmental movements, drawing upon their own diverse models of nature, and collaborating with Western and Chinese environmentalists (Yeh 2014a), although these alliances have likely declined since widespread demonstrations in 2008 and a series of self-immolations since 2011. Our research, although suggesting emergent engagement with global environmentalism, highlights the danger of overwriting complex and diverse religious and cultural practice with externally generated representations or abstract ideas in religious philosophy. It suggests that a blueprint model of sacred sites or indigenous nature conservation should not be imposed, but instead that local people should be able to negotiate and engage in the terms for conservation and natural resource management themselves.

## References

[CR1] Buffetrille K, McKay A (1998). Reflections on pilgrimages to sacred mountains, lakes and caves. Pilgrimage in Tibet.

[CR2] Coggins C, Hutchinson T (2006). The Political Ecology of Geopiety: Nature Conservation in Tibetan Communities in Northwest Yunnan. Asian Geographer.

[CR3] Conservation International (2012). Biodiversity hotspots: Mountains of Southwest China. [WWW document]. URL http://www.conservation.org/where/priority_areas/hotspots/asia-pacific/Mountains-of-Southwest-China/Pages/default.asp.

[CR4] Da Col G (2007). The View From Somewhen: Evenemental Bodies and the Perspective of Fortune Around Khawa Karpo, a Tibetan Sacred Mountain in Yunnan. Inner Asia.

[CR5] Durkheim E, Lambek M (2002). The elementary forms of religious life. A Reader in the Anthropology of Religion.

[CR6] Fischer AM (2005). State Growth and Social Exclusion in Tibet: Challenges of Recent Economic Growth.

[CR7] Goldstein MC, Goldstein MC, Kapstein MT (1998). Introduction. Buddhism in Contemporary Tibet: Religious Revival and Cultural Identity.

[CR8] Gross R, Tucker ME, Williams DR (1997). Buddhist resources for issues of population, consumption and the environment. Buddhism and Ecology: The Interconnection of Dharma and Deeds.

[CR9] Huber T, Korom FJ (1997). Green Tibetans: a brief social history. Tibetan Culture in the Diaspora.

[CR10] Huber T, Huber T (1999). Putting the gnas back into gnas-skor. Sacred Spaces and Powerful Places in Tibetan Culture.

[CR11] Huber T (1999). The Cult of Pure Crystal Mountain: Popular Pilgrimage and Visionary Landscape in Southeast Tibet.

[CR12] Huber, T. (2005). Antelope Hunting in Northern Tibet: Cultural Adaptations to Animal Behaviour. Wildlife and Plants in Traditional and Modern Tibet: Conceptions, Exploitation and Conservation. Memorie della Società Italiana di Scienze Naturali e del Museo Civico di Storia Naturale di Milano, Italy, 33(1):5–17.

[CR13] James SP (2006). Buddhism and the Ethics of Species Conservation. Environmental Values.

[CR14] Keown D (1992). The Nature of Buddhist Ethics.

[CR15] Litzinger R (2004). The Mobilization of ‘Nature’: Perspectives from Northwest Yunnan. The China Quarterly.

[CR16] Makley CE (2007). The Violence of Liberation: Gender and Tibetan Buddhist Revival in Post-Mao China.

[CR17] Makley CE (2013). The Politics of Presence: Voice, Deity Possession, and Dilemma of Development Among Tibetans in the People’s Republic of China. Comparative Studies in Society and History.

[CR18] McNally CA (2004). Sichuan: Driving Capitalist Development Westward. The China Quarterly.

[CR19] Mills MA (2003). Identity, Ritual and State in Tibetan Buddhism: the Foundations of Authority in Gelukpa Monasticism.

[CR20] Mills MA (2006). King Srong btsan sGam po’s Mines: Wealth Accumulation and Religious Asceticism in Buddhist Tibet. Tibet Journal.

[CR21] Mumford S (1989). Himalayan Dialogue: Tibetan Lamas and Gurung Shamans in Nepal.

[CR22] Ogyen Trinley Dorje, H.H. the 17th Karmapa (2011). Walking the Path of environmental Buddhism Through Compassion and Emptiness. Conservation Biology.

[CR23] Ormsby AA, Bhagwat SA (2010). Sacred Forests of India: A Strong Tradition Of Community-Based Natural Resource Management. Environmental Conservation.

[CR24] Parkes G, Tucker ME, Williams DR (1997). Voices of the mountains, trees and rivers: Kukai, Dogen and a Deeper Ecology. Buddhism and Ecology: The Interconnection of Dharma and Deeds.

[CR25] Pirie F (2006). Secular Morality, Village Law, and Buddhism in Tibetan Societies. Journal of the Royal Anthropological Institute.

[CR26] QSR International (2010). NVivo (Version 9). QSR International.

[CR27] Ramble C (1990). How Buddhist are Buddhist Communities? The Construction of Tradition in Two Lamaist Villages. Journal of the Anthropological Society of Oxford.

[CR28] Ramble C, Huber T (1999). The politics of sacred space in Bon and Tibetan popular tradition. Sacred Spaces and Powerful Places in Tibetan Culture.

[CR29] Ramble C (2008). The navel of the Demoness: Tibetan Buddhism and Civil Religion in Highlight Nepal.

[CR30] Richardson HE (1984). Tibet and Its History.

[CR31] Salick J, Amend A, Anderson D, Hoffmeister K, Gunn B, Zhendong F (2007). Tibetan Sacred Sites Conserve Old Growth Trees and Cover in the Eastern Himalayas. Biodiversity and Conservation.

[CR32] Salick, J., Byg, A., and Bauer, K. (2012). Contemporary Tibetan Cosmology of Climate Change. Journal for the Study of Religion, Nature and Culture 6(4): 447–476.

[CR33] Samuel G (1993). Civilized Shamans: Buddhism in Tibetan Societies.

[CR34] Schumacher, E. F. (1966). Buddhist Economics. [WWW document]. URL http://neweconomicsinstitute.org/buddhist-economics.

[CR35] Shen X, Li S, Chen N, Li S, McShea WJ, Lu Z (2012). Does Science Replace Traditions? Correlates Between Traditional Tibetan Culture and Local Bird Diversity in Southwest China. Biological Conservation.

[CR36] Spiro ME (1970). Buddhism and Society.

[CR37] Tenzin Gyatso, the 14th Dalai Lama. (1993). The sheltering trees of interdependence: Buddhist monks reflections on ecological responsibility. [WWW document] URL http://dalailama.com/messages/environment/buddhist-monks-reflections.

[CR38] Tomalin E (2009). Biodivinity and Biodiversity: The Limits to Religious Environmentalism.

[CR39] Tucci G (1988). The Religions of Tibet.

[CR40] Wenbin P, McKay A (1998). Tibetan pilgrimage in the process of social change: the case of Jiuzhaigou. Pilgrimage in Tibet.

[CR41] Weckerle CS, Huber FK, Yang YP, Sun WB (2006). Plant Knowledge of the Shuhi in the Hengduan Mountains, Southwest China. Economic Botany.

[CR42] Wild, R., and McLeod, C. (2008). Sacred natural Sites: guidelines for protected area managers. IUCN, Gland, Switzerland. [WWW document] URL http://data.iucn.org/dbtw-wpd/edocs/PAG-016.pdf.

[CR43] Winkler D (2008). Yartsa Gunbu (Cordyceps sinensis) and the Fungal Commodification of Tibet’s Rural Economy. Economic Botany.

[CR44] White L (1967). The Historical Roots of Our Ecological Crisis. Science.

[CR45] Woodhouse E, McGowan P, Milner-Gulland EJ (2014). Fungal Gold and Firewood on the Tibetan Plateau: Examining Access to Diverse Ecosystem Provisioning Services Within a Rural Community. Oryx.

[CR46] Yeh, E. T. (2014a). The Rise and Fall of the Green Tibetan: Contingent Collaborations and the Vicissitudes of Harmony. In Yeh, E. T., and Coggins, C. (eds.), Mapping Shangrila: Contested Landscapes in the Sino-Tibetan borderlands. University of Washington Press, Seattle, pp. 255–278.

[CR47] Yeh ET, Van der Veer P, Miller J, Smyer Yu D (2014). The rise and fall of the green Tibetan: contingent collaborations and the vicissitudes of harmony. Religious Diversity and Ecological Sustainability in China.

